# Two-Dimensional Frontier-Based Viewpoint Generation for Exploring and Mapping Underwater Environments

**DOI:** 10.3390/s19061460

**Published:** 2019-03-25

**Authors:** Eduard Vidal, Narcís Palomeras, Klemen Istenič, Juan David Hernández, Marc Carreras

**Affiliations:** 1 Underwater Robotics Research Center (CIRS), Computer Vision and Robotics Institute (VICOROB), Universitat de Girona, 17003 Girona, Spain; npalomer@silver.udg.edu (N.P.); klemen.istenic@gmail.com (K.I.); marc.carreras@udg.edu (M.C.); 2 Department of Computer Science, Rice University, Houston, TX 77005, USA; juandhv@rice.edu

**Keywords:** autonomous underwater vehicle (AUV), robotic exploration, view planning (VP), motion planning, frontier-based (FB) exploration, next-best-view (NBV)

## Abstract

To autonomously explore complex underwater environments, it is convenient to develop motion planning strategies that do not depend on prior information. In this publication, we present a robotic exploration algorithm for autonomous underwater vehicles (AUVs) that is able to guide the robot so that it explores an unknown 2-dimensional (2D) environment. The algorithm is built upon view planning (VP) and frontier-based (FB) strategies. Traditional robotic exploration algorithms seek full coverage of the scene with data from only one sensor. If data coverage is required for multiple sensors, multiple exploration missions are required. Our approach has been designed to sense the environment achieving full coverage with data from two sensors in a single exploration mission: occupancy data from the profiling sonar, from which the shape of the environment is perceived, and optical data from the camera, to capture the details of the environment. This saves time and mission costs. The algorithm has been designed to be computationally efficient, so that it can run online in the AUV’s onboard computer. In our approach, the environment is represented using a labeled quadtree occupancy map which, at the same time, is used to generate the viewpoints that guide the exploration. We have tested the algorithm in different environments through numerous experiments, which include sea operations using the Sparus II AUV and its sensor suite.

## 1. Introduction

AUV Autonomous underwater vehicles (AUVs) have become a fundamental tool to perform many underwater tasks, such as close inspection of structures [1], near-bottom surveys [2], or intervention [3]. The use of AUVs has many advantages over alternative technologies such as remotely operated vehicles (ROVs). For instance, the lack of an umbilical cable increases the freedom of movement of AUVs, allowing missions to take place in complex scenarios with high relief or complex artificial structures, where the umbilical cable could get entangled. Furthermore, AUVs require less human intervention allowing for potentially cheaper sea operations. Providing AUVs with the ability to carry out tasks autonomously is a challenge. When the target is in areas with a high level of relief, current algorithms have significant limitations. Our proposal focuses on enabling the use of AUVs in these challenging cases for inspection and mapping purposes. In this work, we present an algorithm which is capable of guiding an underwater robot to obtain a map of a region of interest. Traditionally, this problem has been studied in two different research fields: (CPP) algorithms are focused on obtaining a trajectory that passes through all regions of an area or volume of interest, using a map which can sometimes be of low accuracy. Robotic exploration algorithms, on the other hand, are designed so that there is no need for a prior map, with the goal of obtaining a map of a completely unknown environment. In most cases, the available information about a particular region of the sea is scarce. Because of that, we have designed our algorithm so that it does not use a prior map of the area of interest. The proposed algorithm is therefore an underwater robotic exploration algorithm. As a consequence, the implementation of the algorithm has to be able to run in the robot’s computer, with limited processing power, in order to guide the robot as the mission progresses. To meet this requirement, our algorithm has been designed with computational efficiency in mind, selecting the best data structures to represent the data so that the operations required by the exploration algorithm can be performed fast enough for online planning. Most robotic exploration algorithms are based on the following ideas:

- *Frontier-based (FB) exploration*. Frontier-based methods guide the exploration by focusing on the regions between known an unknown space. This idea was first proposed by Yamauchi [4]. The exploration is guided according to interesting regions in the map. However, the sensor fields of view (FOV) is usually not taken explicitly into account. Furthermore, if the target frontier is the boundary between known and unknown space, as done in the original and many other publications, the robot has a tendency to navigate in a straight line exploring as much as possible until something is reached. This behavior is desirable for indoor exploration, but it is not appropriate for underwater exploration because the robot will only explore open water unless some limits are specified.
- *View planning (VP)*. View planning algorithms evaluate different candidate viewpoints to determine the actions that the robot must perform. A viewpoint is commonly defined as a particular configuration of robot/sensors. When performing CPP the best route that explores all viewpoints is commonly found by solving a variant of the art gallery problem (AGP) and the traveling salesman problem (TSP). In contrast, robotic exploration algorithms based on VP usually use the next-best-view (NBV) approach, where the next best viewpoint is planned online according to the current map and robot location. The first example of VP using the NBV approach was developed by Connolly [5]. One of the advantages of VP algorithms is that they are explicitly aware of the sensor FOV. However, since usually there is an infinite amount of possible viewpoints it is difficult to select them for their evaluation. For this reason, it is common to generate the viewpoints randomly or to reduce the amount of possible viewpoints according to the specific problem. Furthermore, to properly evaluate a viewpoint it is sometimes necessary to use a ray-casting approach, which might be too slow for online computation.
- *Reactive algorithms (RAs)*. Reactive algorithms, such as control-based approaches, can also be used for robotic exploration as done in McEwen et al. [6]. Even potential fields can be used for robotic exploration [7]. They provide a simple framework which is easy to implement, but they suffer from local minima problems, and it is difficult to precisely account for the FOV of the sensors during planning.

The proposed approach combines the strengths of FB exploration and VP methods to obtain an algorithm specifically tailored for underwater robotic exploration. The frontiers extracted from the map are used to deterministically generate viewpoints for exploration. By considering frontiers between explored and unexplored areas, and between seen and unseen areas, data continuity and overlap is imposed, which is good for mapping purposes because it enables feature-matching and data registration between scans. A requirement of our proposed method is that the explored structure must have vertical relief. Then, the exploration is performed in a 2D slice at a user defined depth. Furthermore, our algorithm is capable of autonomously guiding an underwater robot to obtain both the occupancy map and the optical data of a region of interest in a single exploration mission. To demonstrate the feasibility of our approach we present simulations and experimental data using the Sparus II AUV. The proposed approach is an extension of our previous work in [8,9]. In this work, several aspects of the viewpoint generation process have been improved, mainly to improve robustness and safety. New experimental data has been obtained in challenging scenarios, and a quantitative evaluation of the obtained results is presented. The remainder of this paper is organized as follows. Section 2 presents a review of important related work to our underwater robotic exploration problem. Then, Section 3 explains the details of the proposed underwater robotic exploration algorithm. Section 4 shows the robotic platform that has been used to generate the experimental outcomes, presented in Section 5. Finally, Section 6 presents the conclusions and evaluates further lines of investigation.

## 2. Related Work

This section presents important related work to our underwater robotic exploration problem. Table 1 summarizes this section presenting a classification of algorithms by the amount of *prior knowledge* used, *domain*, *dimensionality* and *approach*.

### 2.1. Methods That Use a Prior Map

In the underwater domain, Galceran et al. [10] presented a 2.5D approach for inspection of complex underwater structures. In their approach, a prior map is used to compute a nominal path that covers all the scene. Then, the robot follows the precomputed path while adapting it to what is perceived in situ, thus allowing some deviation to account for the navigation drift and inaccuracies in the prior map. Recently, Palomeras et al. [11] presented a VP algorithm which samples viewpoints from a previous model and then solves the TSP. In their work, simultaneous localization and mapping (SLAM) is used during the mission to ensure minimal deviations with respect to the previously planned trajectory. However, results were supported by simulations only.

Regarding other domains, Blaer and Allen [12] presented a two stage VP approach for 3-dimensional (3D) site modeling with unmanned ground vehicles (UGVs). In their initial stage, a minimal set of views is planned in 2D to cover a prior map of the scene, and then, in a second stage, the resulting model is improved by considering 3D views of the 3D model obtained in the first stage. Bircher et al. [13] presented a VP algorithm for structural inspection using unmanned aerial vehicles (UAVs). Their method employs an alternating two-step optimization to find viewpoints for coverage while reducing the path cost.

All the aforementioned methods can be used when a prior map is available. Although they share some similarities with the methods in the following section, they are not directly applicable to our problem since we do not have a prior map of the area to be explored.

### 2.2. Methods That Do Not Use a Prior Map

In the underwater domain, the robotic exploration literature is scarce. Aside from our previous VP work in Vidal et al. [8] and Vidal et al. [9], Williams et al. [14] proposed a target reinspection method for AUVs equipped with a synthetic aperture sonar (SAS). In their approach, after a first constant altitude mission, locations of potential interest are automatically inspected before the vehicle surfaces, which can be considered a form of VP exploration. However, the initial constant altitude mission can only be performed if the area does not contain 3D relief, so it is not suitable to our exploration problem. Regarding 3D environments, Kim and Eustice [15] and Hover et al. [1] developed VP techniques for ship hull inspection. While a prior rough map was necessary to plan the path to explore the propellers and rudders, the rest of the hull was inspected without a prior model. The inspection follows a preplanned lawn-mover trajectory that is merged with target revisiting. This approach is very specific and it is not directly applicable to our exploration problem. McEwen et al. [6] presented a reactive and control-based approach where an iceberg was mapped by performing several autonomous wall following missions at different depths. This approach can not be directly applied to our problem because we can have multiple objects with high relief (for instance, our breakwater blocks scenario).

Some of the methods that are used for object reconstruction can also be adapted for robotic exploration. Connolly [5] proposed the NBV methodology to autonomously plan views to reconstruct a 3D object. In the same line, Vasquez-Gomez et al. [16] presented a NBV algorithm to model arbitrary objects in 3D, and Vasquez-Gomez et al. [17] refined the method by adding uncertainties. Their method does not need prior knowledge regarding the shape of the object, but information about its size and location is required. Isler et al. [18] also developed a NBV uncertainty-aware approach for active volumetric 3D reconstruction. Although the aforementioned 3D reconstruction methods cannot be directly applied to underwater exploration, our algorithm is based on ideas developed in these methods, such as the NBV methodology, so they are relevant to our work. 

Regarding exploration algorithms for ground vehicles, Yamauchi [4] initially proposed the FB method for 2D robotic exploration. González- Baños and Mao [19] applied NBV strategies to robotic exploration by planning randomized views that maximize information gain over a polygonal model of the environment. Burgard et al. [20] explored FB methods and even extended them to work with multiple robots. Then, Fox et al. [21] proposed a distributed multirobot exploration algorithm for ground vehicles where the robots actively verify their relative locations with the goal of improving the map consistency. Finally, Stachniss et al. [22] proposed the exploration of unknown indoor environments using a team of mobile robots. Their method uses a classifier to assign labels to different locations in the map, and then these labels are used to guide the exploration using a utility function.

In the aerial domain, Schmid et al. [23] presented a two step process where first a coarse digital surface (DSM) of the environment is built, and then viewpoints are planned to acquire the data for a 3D reconstruction. Yoder and Scherer [24] presented a FB algorithm for micro aerial vehicles (MAVs). In their approach, the different viewpoints are evaluated according to the visibility of frontier cells, determined by ray-tracing. Finally, Bircher et al. [25] and Papachristos et al. [26] proposed a method based on the rapidly-exploring random tree (RRT) to perform exploration without a prior map. A random tree is generated and the best branch is chosen according to the information gain, measured by the amount of mapped and unmapped cells visited when following the generated viewpoints in the branch.

Our algorithm combines different aspects from the presented related work. Furthermore, we extend existing approaches by considering coverage of two sensors simultaneously in a single exploration mission. To the best of the authors knowledge, this is the first underwater exploration algorithm that has this capability.

## 3. Frontier-Based Viewpoint Generation Method for Exploration

The proposed 2D robotic exploration method seeks full coverage of the environment with two different types of data:

- Occupancy data: a mechanically scanning profiling sonar is used to obtain occupancy data from the environment. This kind of sonar sensors mechanically rotate a narrow acoustic beam in order to measure ranges from different orientations. Since the beam rotates along one axis, the field of view covers a user defined sector from a plane. A scan usually takes several seconds to be obtained.
- Optical data: a camera acquires images from the environment. The exploration algorithm does not use the images so no live feedback from the camera is required. Only an estimation of its FOV is used for exploration planning purposes.

**Figure 1:**
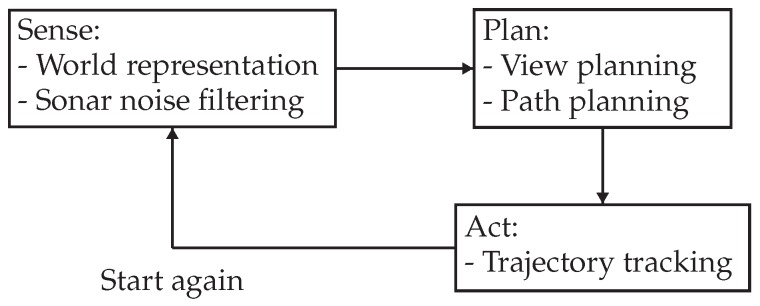
Each part of the proposed algorithm is associated to the corresponding task in the hierarchical/deliberative robotic paradigm.

The algorithm has been designed to fit a hierarchical/deliberative robotic paradigm where, according to Arkin [27], the tasks that the robot iteratively performs can be classified in three categories: *sense*, *plan* and *act* (see Figure 1).

In the remainder of this section the different parts of the proposed method will be described.

### 3.1. World Representation (Sense)

Using the data received from the sonar sensor, and considering the FOV of the camera, our approach creates a labeled grid map to represent and encode the information perceived from the environment. The different possible cell labels are:

- Unknown cells. The environment is initially assumed to be unknown. Thus, this is the initial state label for all cells in the map.
- Empty cells. They represent collision-free areas where the vehicle can navigate.
- Occupied cells. They correspond to the areas where the profiling sonar has detected an obstacle. They represent walls and objects in the environment.
- Viewed cells. The occupied cells that have been inside the camera FOV are labeled as viewed.
- Range candidate cells. The unknown cells that are next to empty and occupied cells are range candidate cells because they represent regions of potential interest to continue the occupancy exploration.
- Camera candidate cells. The occupied cells that are next to empty and viewed cells are camera candidate cells because they represent the areas that should be optically explored.

**Figure 2:**
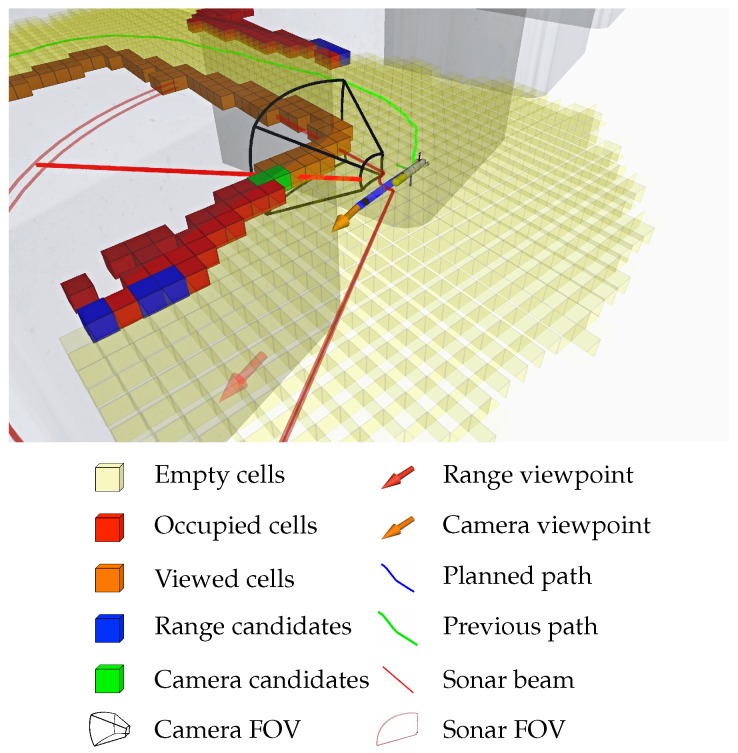
This figure shows all possible cell labels in a single exploration picture. The FOVs of the sensors are also shown.

**Figure 3:**
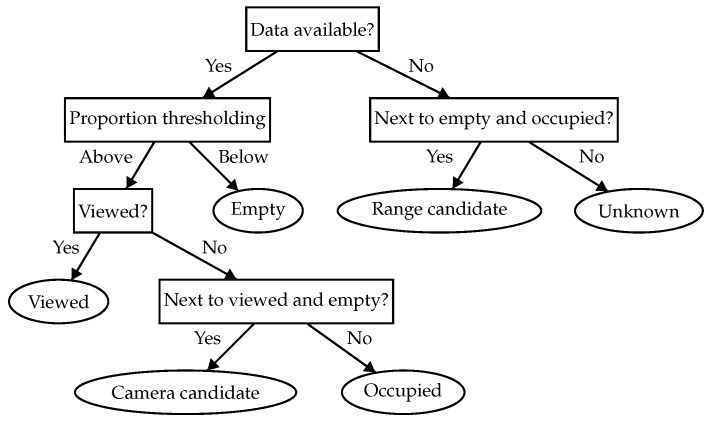
Map generation algorithm. After following the algorithm, a cell is classified and a label is obtained (leafs). When new measurements are received for a cell, the algorithm reevaluates its new label.

Figure 2 depicts all labels in a single exploration capture. When new data is received from the sonar, the cell logic diagram represented in Figure 3 is followed to determine the label that each cell is given. The label of a cell can change several times during a mission. For instance, a cell that was initially given the occupied label might become empty if it receives enough empty measurements from the sonar (this behavior is represented by the *proportion thresholding* node in the diagram of Figure 3).

**Figure 4:**
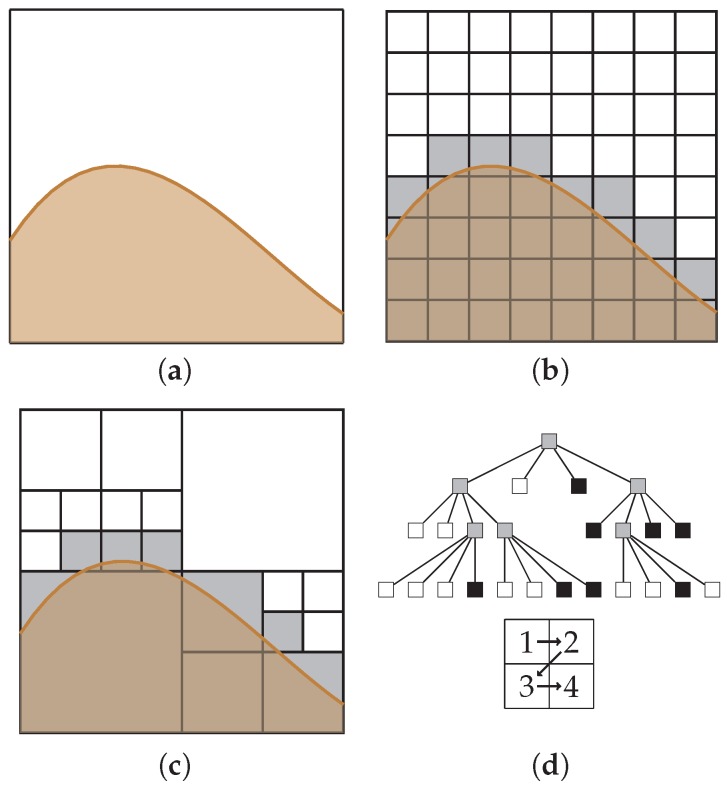
Example of a quadtree data structure: (**a**) the structure to represent; (**b**) a rasterized version of the structure, where the space represented using equally sized cells; (**c**) recursive subdivision of the space to represent the occupancy as a quadtree; and (**d**) the corresponding tree.

One of the novelties of our proposed algorithm is that the grid map is internally stored in several quadtrees. A quadtree is a space partitioning tree-based data structure which recursively subdivides each node to exactly four children (see Figure 4). This data structure enables some operations to be performed efficiently, such as:

- Nearest neighbors and k-nearest neighbors queries. For any specific target cell, it is possible to find the nearest cell or cells of a particular label.
- Range queries. For any specific target cell, it is possible to find all the cells within a certain distance for cells of a particular label.

In our approach, several quadtrees are used so that the previous operations can be performed to the required cell labels in isolation, and we take advantage of this in the viewpoint generation process.

There exist public implementations of such tree data structures. For instance, the Octomap framework from Hornung et al. [28] implements an octree data structure (3D equivalent of a quadtree) and it is common in the robotic community. However, at the time of this publication, Octomap does not provide an implementation of nearest neighbor and range queries. To overcome this limitation, we have implemented our own quadtree data structure. 

Finally, our map representation can be easily adapted to 3D environments by using an octree data structure instead of a quadtree. The rest of the operations, such as the computation of the surface normal and queries to the tree, are also well defined in a 3D space.

### 3.2. Sonar Noise Filtering (Sense)

Underwater sonar sensors suffer from different kinds of noise, which can potentially corrupt the map created from such data. Our robotic exploration algorithm relies on sonar data to determine what are the next best actions to take for exploration, so it is important to minimize the negative effects of the sonar noise.

When a sonar measurement is obtained, we first apply some basic filtering, which discards data in several situations:

- When the measurement is close to the minimum and maximum range of the sensor.
- When the measurement corresponds to a location near the water surface.
- When the vehicle is not stable or moving fast.

**Figure 5:**
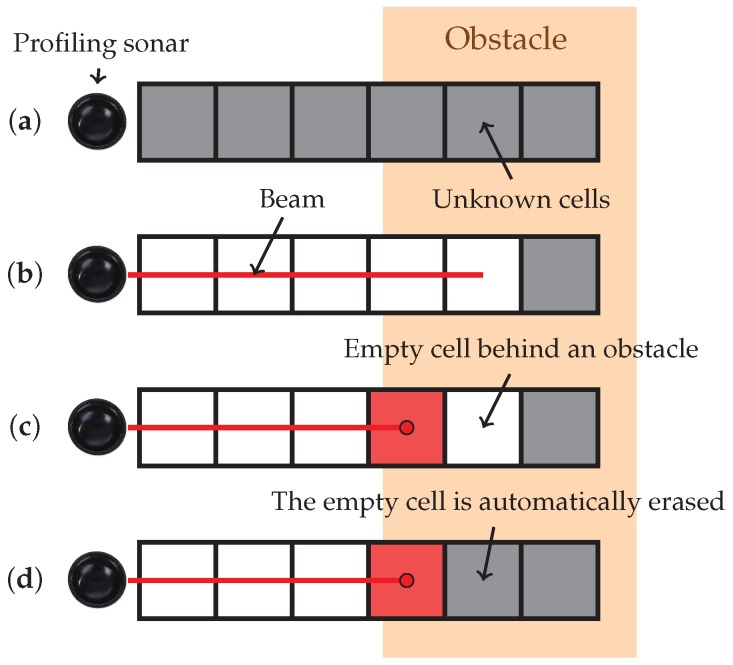
If not accounted for, false negatives can affect the map consistency. Consider the following sequence of events: (**a**) initially all cells have unknown state; (**b**) a false negative is received, resulting in empty cells along the beam until the maximum range of the sensor; and (**c**,**d**) finally a correct measurement is received. If each cell is considered independently, this sequence of events leaves empty cells behind the occupied cell (**c**). With our approach, this situation is detected and empty cells behind the obstacle are automatically erased (**d**) so that empty space is consistent with all occupied measurements.

After basic filtering has been performed, the measurement is incorporated into the map according to the strategy we defined in Vidal et al. [9], which improves the map consistency when false negatives are present. If the right combination of sensor measurements is received, empty space can appear behind obstacles, as depicted in Figure 5. Our approach is able to overcome this problem and generates coherent maps even when false negatives are received. The basic idea behind the false negative noise rejection algorithm is that empty measurements can only come from nearby empty cells, so when a cell changes its state from empty to a different state, neighboring empty cells must be reevaluated.

### 3.3. View Planning (Plan)

Once the data from the sensors has been incorporated into our map, the next step is to generate viewpoints at locations that allow the exploration to continue. The proposed view planning strategy has been designed so that it takes advantage of the efficient operations allowed by our map representation. This is key to achieve the required performance to enable online missions. 

Two different types of viewpoints are generated:

- *Range viewpoints.* Each range candidate cell in the map potentially generates a range viewpoint. Range viewpoints allow the exploration of the environment using the scanning profiling sonar, as they are focused on the frontier between occupied and unknown regions.
- *Camera viewpoints.* Camera candidate cells represent the frontier between optically explored and unexplored areas, and they potentially generate camera viewpoints.

**Figure 6:**
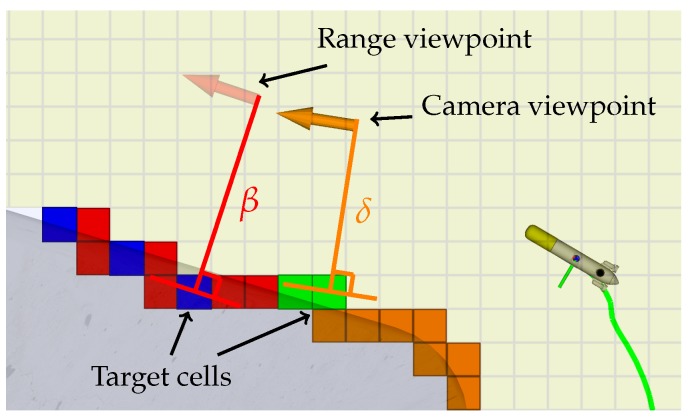
Viewpoint generation example. Each target cell generates a viewpoint at a user configurable distance (in this case, β and δ) along the estimated surface normal.

Figure 6 depicts an example of the viewpoint generation process. To generate a viewpoint from a candidate cell the following deterministic procedure is followed:

- The surface normal is computed using as a reference the occupied and viewed cells around the candidate cell.
- The viewpoint is placed along the surface normal at a user defined distance, which must account for the sensor FOV.
- If the generated viewpoint is inside an empty cell it is stored for further evaluation. Otherwise, it is discarded.
- If the generated viewpoint is too close to the obstacles it is discarded. Otherwise, it is considered a safe viewpoint. Due to safety concerns, in this work, the concept of safe viewpoints is more strict than in [8,9].

The fact that the viewpoint generation process is deterministic is good for repeatability and overall understanding of the exploration maneuvers.

Once the set of all safe viewpoints has been computed, the viewpoints are evaluated according to a cost function, which captures how far a viewpoint is with respect to the current robot configuration. At this stage, both range and camera viewpoints are considered without prioritizing one over the other. Unfortunately, solving a complete path planning problem for each viewpoint is not possible online due to computational time constraints. Alternatively, the proposed cost function uses a weighted Euclidean distance which additionally accounts for the difference in orientation at the beginning and at the end of the path. While in [8,9] the weighting factor had to be manually chosen, in this work it is automatically computed using the maximum surge velocity and maximum yaw turning rate. Once all viewpoints have been evaluated, the viewpoint with the lowest cost value is selected. The cost function is described by Equations (1) and (2):

(1) $$\beta =atan2(p_{y}-q_{y},p_{x}-q_{x})$$

(2) $$cost\left(q,p\right)=∥p_{xy}-q_{xy}∥+\frac{v_{max}}{{\dot{\theta }}_{max}}\;\left(\left|\mathrm{wrap}\left(\beta -q_{\theta }\right)\right|+\left|\mathrm{wrap}\left(p_{\theta }-\beta \right)\right|\right)$$

where *q* represents the robot configuration, *p* represents the viewpoint configuration, $v_{max}$ is the maximum surge velocity, ${\dot{\theta }}_{max}$ is the maximum turning rate and wrap() converts an angle to a value contained within the range (−π,π]. Finally, the algorithm stops the exploration when there are no more candidate viewpoints or when a timeout has expired.

### 3.4. Path Planning (Plan)

After computing the next best viewpoint, the robot has to navigate from its current configuration to the selected viewpoint, while avoiding the obstacles present in the current map. To generate such trajectories, we propose the use of the asymptotic optimal rapidly-exploring random tree (RRT*) path planner.

Since the robotic exploration algorithm runs on the robot’s computer, with limited computational resources, we have simplified the planning problem to compute paths in a 2D configuration space, where a configuration contains only the position of the robot. Considering the orientation of the vehicle in the path planning would significantly slow down the planner, making it unsuitable for online purposes. At the same time, safety can be preserved by checking whether the smallest possible circular area containing the robot is colliding with the obstacles in the map (thus ensuring the state is valid in any possible orientation).

**Figure 7:**
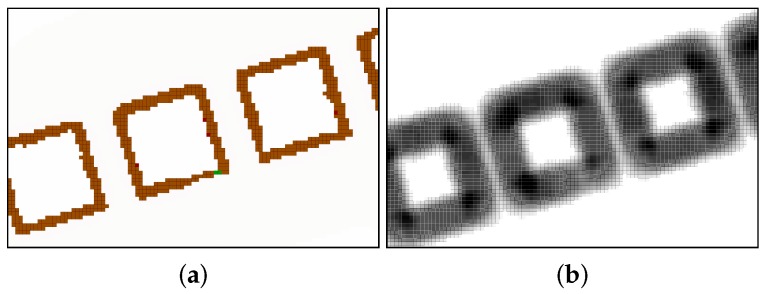
Correspondence between a real map and its risk value map. The real map is displayed in (**a**). In (**b**) the risk is displayed using a gradient from white to black color (white represents the lowest risk and black represents the highest risk). The highest risk appears near the walls of the obstacles.

In our implementation, the path planner optimizes the integral of a risk function along the path. The risk associated with a particular state reflects how close it is to the obstacles in the map. Therefore, the risk is high next to obstacles and lessens as the distance increases. Figure 7 visually represents the risk cost in a particular map example.

The risk function is described by the following equation:

(3) $$risk\left(M,q,r\right)=1+\psi^{2}O\left(M,q,r\right)$$

where *M* represents our labeled quadtree-based grid map, *q* represents the robot configuration, ψ represents the map resolution and O(M,q,r) returns the amount of occupied cells around the given configuration *q* up to a distance *r*.

By optimizing the integral of the risk we achieve two goals simultaneously:

- Shorter paths are preferred.
- Paths that navigate far from the obstacles are preferred.

### 3.5. Trajectory Tracking (Act)

Once the path that allows the vehicle to reach the selected viewpoint has been computed, a line of sight (LOS) trajectory tracking controller [29] is used to follow it with minimum error. When the vehicle approaches the target viewpoint, the trajectory tracking controller is stopped and the vehicle is oriented according to the orientation of the viewpoint. Due to the thrusters configuration in the robot and the trajectory tracking controller used, lateral currents can affect the trajectory tracking performance. However, the control problem with water currents is out of the scope of this work. From our experimental experience, the selected approach can operate with lateral currents of up to 0.3 m/s, which is sufficient for the autonomous tasks shown in this work.

### 3.6. Summary of the Algorithm

Algorithm 1 summarizes the proposed robotic exploration approach. Lines 3 to 6 represent Section 3.1 and Section 3.2. Lines 7 to 11 correspond to Section 3.3 and line 12 corresponds to Section 3.4. Finally, Section 3.5 is represented by line 13.

 **Algorithm 1:** Exploration methodology  **Input**: Range measurements, robot position.  **Output**: Exploration trajectory, map. 
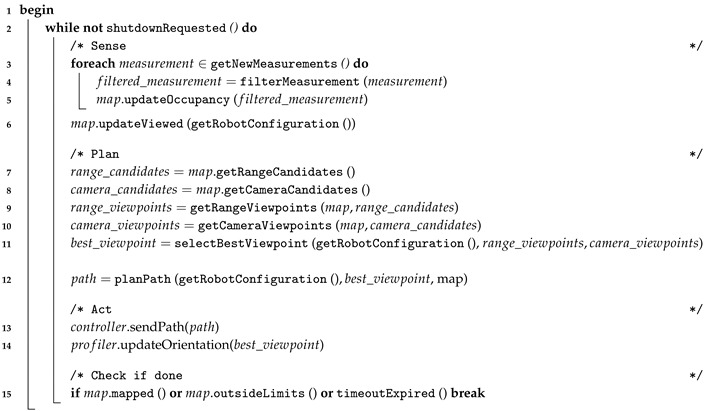


**Figure 8:**
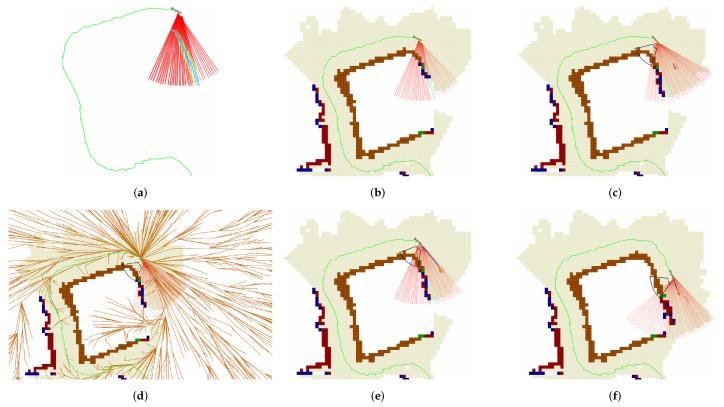
Sequence of operations performed by the proposed robotic exploration algorithm: (**a**) Initially the robot receives data from the sonar sensor. (**b**) The data is incorporated into the map. (**c**) The best view is selected. (**d**) A safe path is computed from the robot configuration to the selected viewpoint. (**e**) The path (blue line) is followed by the trajectory tracking controllers. (**f**) Finally, the robot reaches the viewpoint. By then, the map has changed and new viewpoints are generated to continue the exploration.

Figure 8 depicts the sequence of operations performed by the proposed exploration algorithm in a particular example.

## 4. Experimental Platform

**Figure 9:**
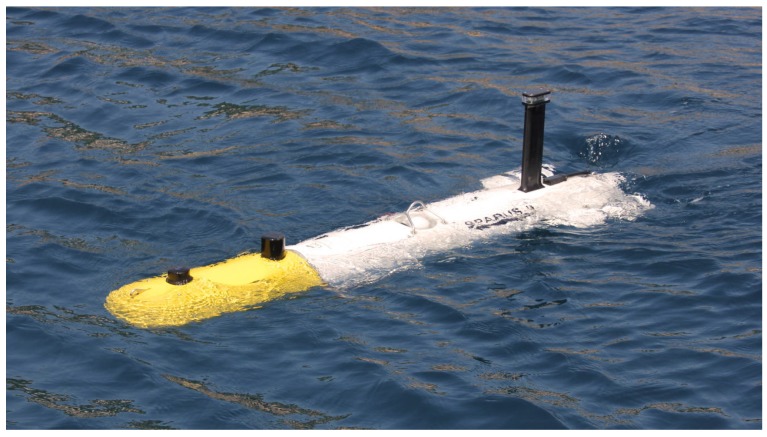
Sparus II AUV, a torpedo-shaped robot with partial hovering capabilities. It has been used to validate our robotic exploration algorithm.

To validate the proposed robotic exploration algorithm we have used the Sparus II AUV (see Figure 9). This robot has two horizontal and one vertical thruster, allowing for partial hovering capabilities. The surge, heave and yaw degrees of freedom (DOFs) are actuated while the sway, roll and pitch DOFs are underactuated. It has a diameter of 0.23 m and it is 1.6 m long. It is rated for a maximum depth of 200 m. It has a 1.4 kWh battery which allows between 8 and 10 h of operation. Regarding the onboard computer, this particular robot has a dual core i7 CPU with 8 Gb of RAM. To estimate its position and orientation, the vehicle has a Doppler velocity log (DVL) sensor, an attitude and heading reference system (AHRS), a pressure sensor, and a global positioning system (GPS) sensor to receive fixes at surface. Further information regarding the vehicle can be found in Carreras et al. [30].

The front part of the vehicle is the payload area, where the cameras and the scanning profiling sonar have been installed.

**Figure 10:**
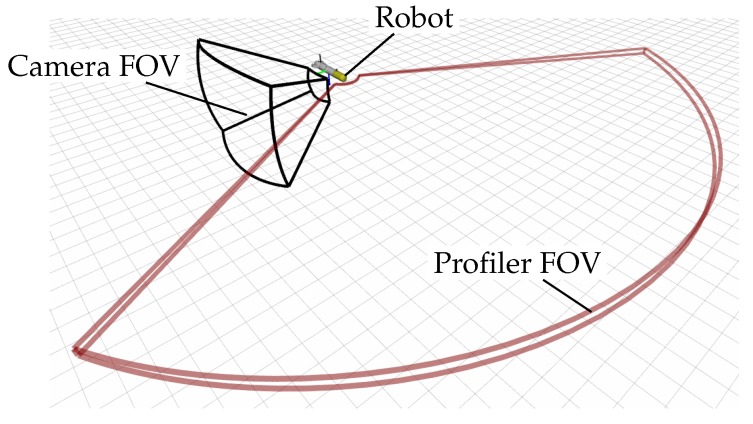
The camera FOV is represented by the black frame (the camera is oriented towards the right side of the vehicle), and the profiling sonar FOV is represented by the red frame (covering mainly the front part of the vehicle).

By means of a mechanically rotating beam, the sonar FOV spans 120 degrees. Although the robot is oriented according to the viewpoint, the FOV of the profiling sonar is also dynamically adjusted during the mission, so that it points towards the exploration target, while always covering the front of the vehicle. Figure 10 shows a representation of the FOV of all sensors.

Throughout this work we have used GoPro Hero 4 Black cameras (GoPro, San Mateo, CA, USA) to acquire the images used for the reconstruction purposes (the optical reconstruction procedure, described in Hernandez et al. [31], is out of the scope of this work, but it is useful for us to demonstrate that the algorithm ensures full optical coverage according to the obtained map). A set of three cameras have been used, positioned at the front of the vehicle and oriented in the right, right-down and forward-right-down directions. Although the exploration algorithm planned the viewpoints for the right oriented camera, the other cameras maximized the optical coverage while maintaining the ability to perform feature matching between the obtained images. No artificial light has been used for the experiments presented in this work, but it could be used during low visibility operations.

**Figure 11:**
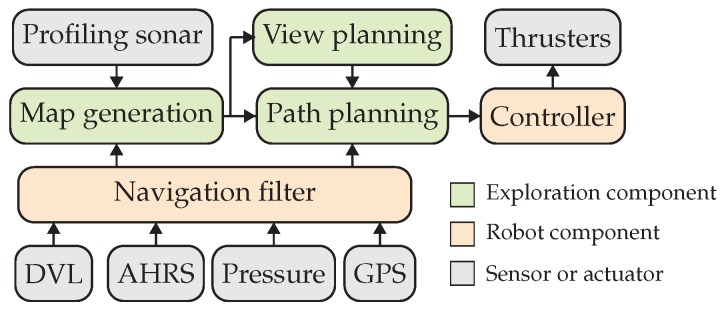
The modular design of our proposal eases integration with typical robotic software architectures. The green blocks are the components developed in our proposal. They interact with the profiling sonar sensor, the vehicle controller and the navigation block. The navigation block is in charge of the localization of the vehicle through dead reckoning, using a Doppler velocity log (DVL) sensor, an attitude and heading reference system (AHRS), a pressure sensor and a global positioning system (GPS) sensor.

The proposed algorithm has been implemented using the C++ programming language, and it has been connected to the rest of the robot’s software architecture using the the robot operating system (ROS) [32]. Figure 11 shows the interconnections between the different parts of the proposed exploration method.

## 5. Experimental Outcomes

The proposed robotic exploration algorithm has been validated in three different scenarios. The first scenario corresponds to a series of breakwater concrete blocks, which provide a challenging testing environment because of its narrow passages. The second scenario is an isolated rock next to the coast cliffs. This natural environment has been used to test the algorithm so that it can explore targets with complex geometry. Finally the algorithm has also been tested at 28 m depth by exploring an underwater seamount. In this section the obtained exploration trajectories and their corresponding 3D optical reconstructions are presented and discussed.

### 5.1. Breakwater Blocks

**Figure 12:**
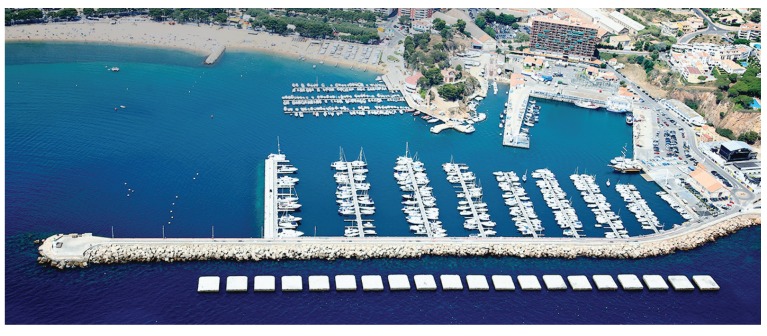
Aerial view of the harbor of St. Feliu de Guíxols. The breakwater blocks can be seen at the bottom part of the image.

**Figure 13:**
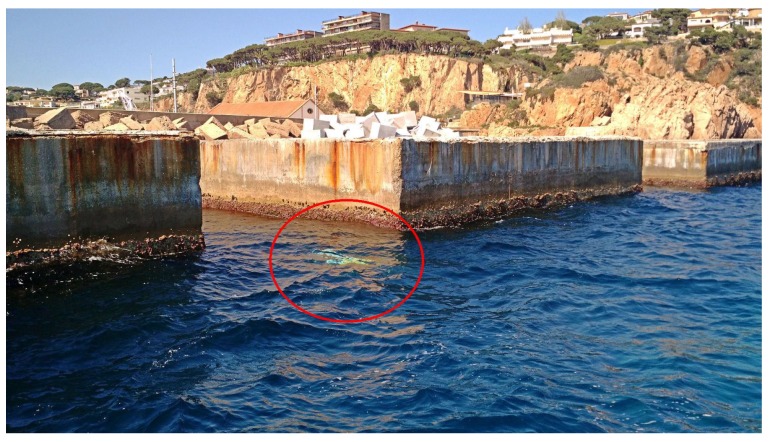
Sparus II AUV performing an autonomous mission in the blocks environment.

The first scenario is a series of breakwater concrete blocks located outside the harbor of St. Feliu de Guíxols, Girona. The size of each block is approximately 12 × 12 m. It is a man-made scenario presenting a simple geometry. However, due to its narrow passages, it is a challenging scenario for underwater exploration. Figure 12 shows an aerial view of this site and Figure 13 shows the Sparus II AUV during an autonomous mission in the breakwater blocks.

**Figure 14:**
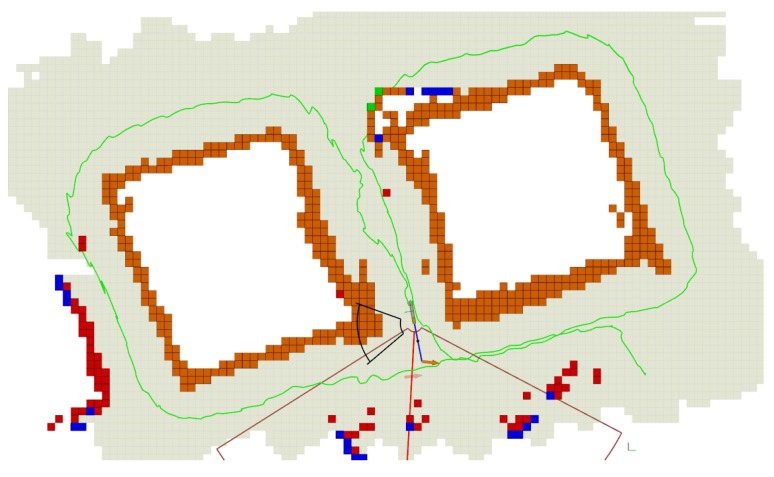
Real inspection of two breakwater concrete blocks. Each block spans an area of approximately 12 × 12 m. The robot trajectory began in front of the block that appears on the right side of the image.

The robot performed the mission at a depth of 1.75 m, allowing for the use of a surface buoy with a Wi-Fi connection, which was used for visualization and safety purposes. The exploration trajectory was about 100 m long and the maximum surge speed was 0.3 m/s. Figure 14 shows the robot trajectory during an autonomous exploration of the breakwater blocks.

**Figure 15:**
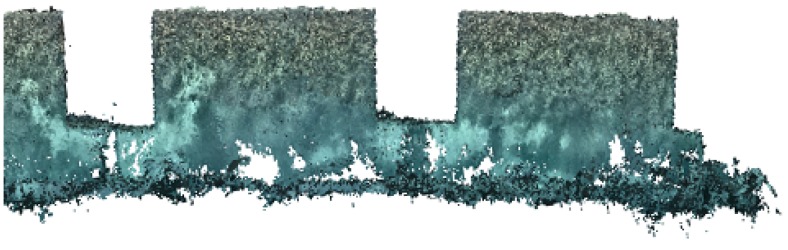
Reconstruction of the breakwater blocks using optical data.

As it can be seen, the robot’s estimated position drifted. The shape of the blocks is distorted and some of the walls appear twice in the map. However, correcting the localization drift is out of the scope of this work. At the same time, localization drift can be assumed to accumulate over time, so is usually low in areas that have been recently explored, and high in areas that have been previously explored and are revisited after some time. Since the vehicle normally operates near areas that have been recently explored, some navigation drift can be tolerated without negatively affecting the performance of the algorithm. Finally, Figure 15 shows the optical reconstruction obtained in the Breakwater blocks scenario.

This scenario has been extensively used to test our previous versions of the the presented approach. In Vidal et al. [8] the robot was able to autonomously explore 8 consecutive blocks. This demonstrated that our method is suitable for man-made structured environments. However, the approach used in [8] had safety issues which caused the robot to navigate too close to the concrete blocks in some circumstances. In this work, only safe viewpoints are used for exploration, leading to safer exploration trajectories.

### 5.2. Punta del Molar

**Figure 16:**
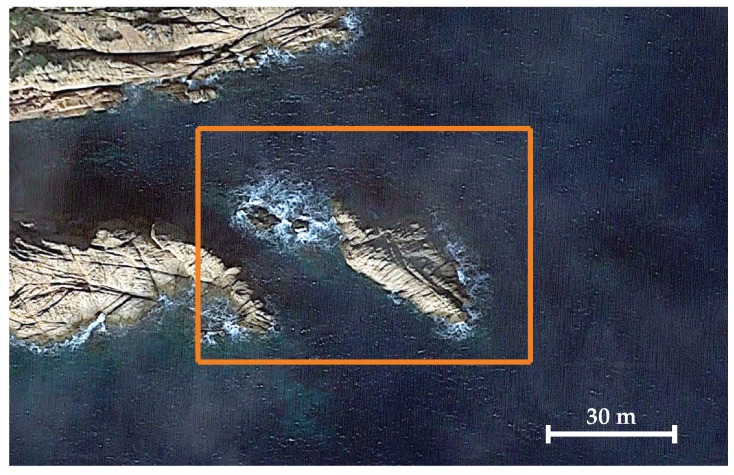
Satellite view of Punta del Molar, *Google Earth, 2017*. The coast cliffs can be seen at the top and left sides of the image.

The second scenario corresponds to an isolated rock located next to the coast cliffs of St. Feliu de Guíxols, Girona. Figure 16 shows a satellite view of this site. The rock is about 60 m with a variable and irregular width.

**Figure 17:**
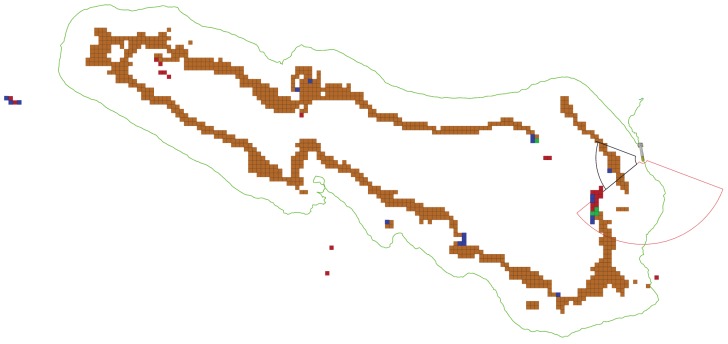
Real experiment showing the inspection of a natural rock surrounded by water near the coast cliffs. The rock is approximately 60 m long (please, see Figure 16). The inspection trajectory ended near the initial point, following the rock clockwise. This is the result of having the cameras mounted pointing towards the right hand side of the robot. In this figure, empty space cells are not represented.

Figure 17 shows the robot trajectory during the exploration of Punta del Molar. This mission was performed at a depth of 2.5 m, also allowing for a safety Wi-Fi buoy. The full exploration took 17 min and the traveled distance was around 170 m.

**Figure 18:**
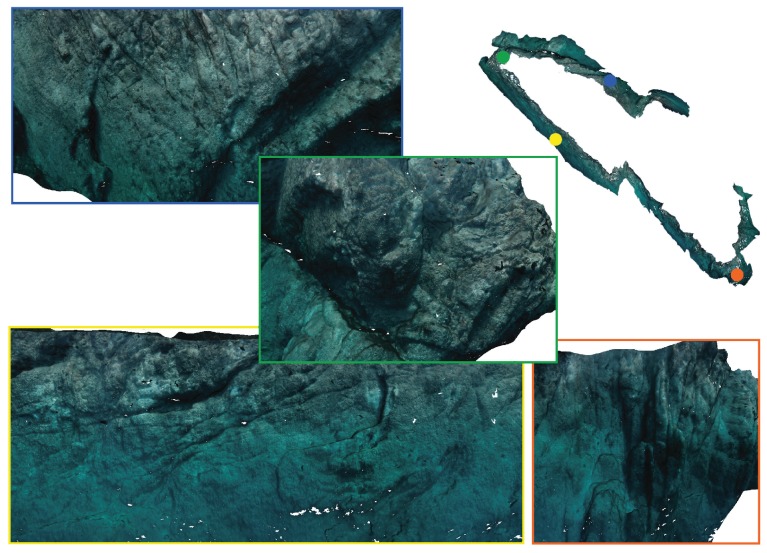
Optical reconstruction of the Punta del molar environment.

For this scenario a 3D reconstruction has also been performed. It is shown in Figure 18. In this case, due to accumulated drift and poor visibility, the optical reconstruction pipeline was not able to close the loop and provide a complete 3D model.

The experiments in this scenario show that the algorithm is suitable for natural unstructured environments.

### 5.3. Amarrador Seamount

The Amarrador seamount is a 12 m high underwater seamount, rising from 40 m depth. Its base spans an area of 15 × 30 m. This natural environment has been used to demonstrate that the algorithm is able to explore targets with complex geometry. Furthermore, it is located in an area with strong currents of up to 0.5 m/s, which makes operations more difficult.

In order to autonomously find the Amarrador seamount (only an approximate GPS position was available) and trigger the exploration algorithm, the AUV performed the following sequence of actions:

1. The robot navigates to the diving location, which is located at a distance from the target.
2. The robot dives to the desired exploration depth.
3. The robot performs a spiral maneuver around the expected underwater boulder location to localize the structure.
4. When the sonar detects the structure, the proposed robotic exploration algorithm is triggered.
5. The exploration finishes once the map is complete or when a timeout has expired.

**Figure 19:**
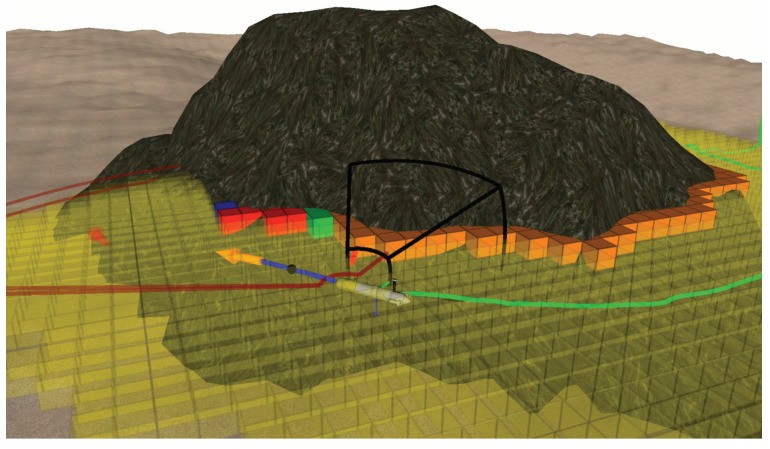
Simulated exploration of the Amarrador seamount.

This sequence of actions is tailored for this specific scenario and it is not part of the presented algorithm. The procedure was first tested in simulation. Figure 19 shows a picture of the robot exploring the seamount in simulation.

**Figure 20:**
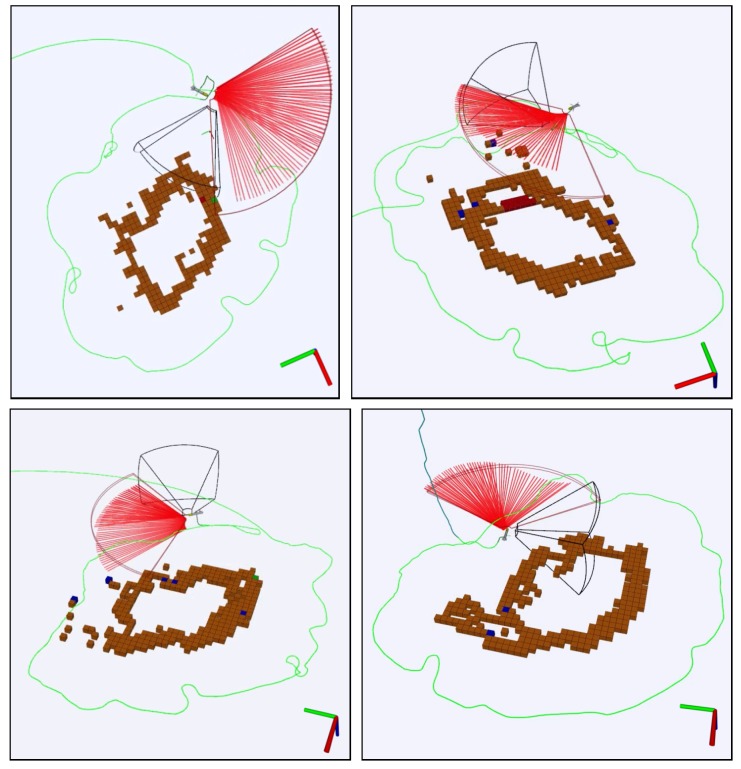
Experimental results in the Amarrador seamount. The four images depict the trajectory of four different successful missions conducted with Sparus II AUV. The robot autonomously explored the underwater seamount in 2D, circumnavigating the rock while keeping the distance suitable for data acquisition. The orientation of each image has been adapted to better visualize the map. Red axis is north, green axis is east, and blue axis is down.

**Figure 21:**
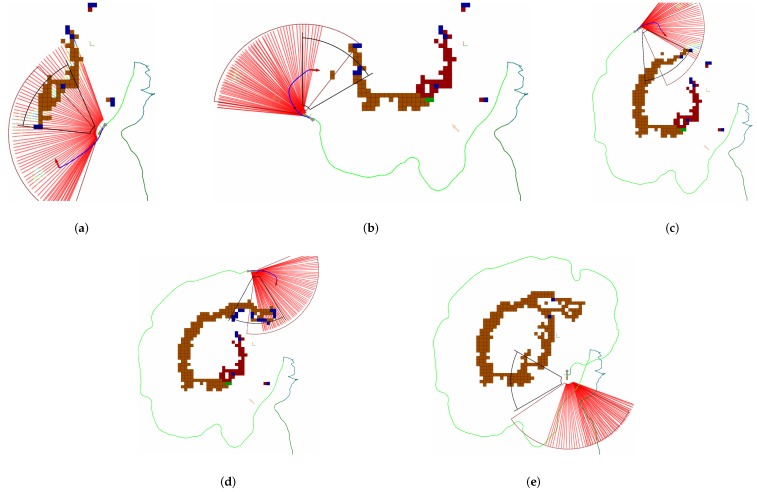
Different captures during a real exploration of the Amarrador seamount. In (**a**) the robot finds the seamount and starts mapping it. Then, in (**b**–**d**) the robot keeps going to the next best view to keep the exploration going. Finally, in (**e**) the robot has a complete map, so no more viewpoints can be generated. The mission is finished.

Then, the approach was tested in real sea experiments. Several autonomous missions were successfully performed using Sparus II AUV. Figure 20 shows different successful exploration missions, and Figure 21 shows the evolution of one of the missions to help understanding the sequence of maneuvers that are performed.

**Figure 22:**
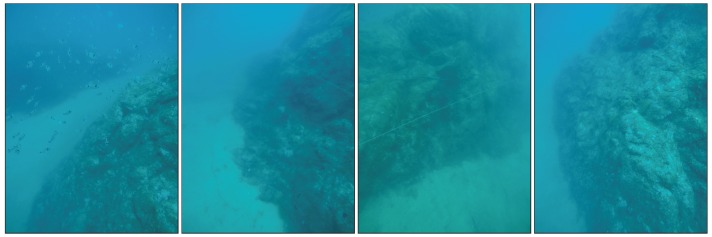
Different images obtained during autonomous exploration missions of the Amarrador underwater boulder. The robot performed the exploration at a depth of 28 m, and the distance between the robot and the rock was 5 m.

**Figure 23:**
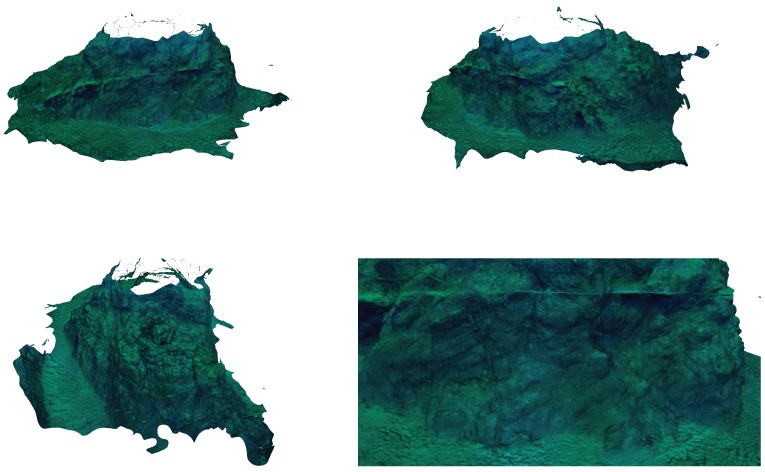
Using the images acquired during a 2D autonomous exploration of the Amarrador seamount, a 3D reconstruction has been obtained. The geometry is presented with the texture extracted from the same images.

Finally, Figure 22 shows different images obtained from the cameras during the autonomous exploration mission, and Figure 23 shows the reconstruction of the Amarrador seamount. The images show the obtained textured 3D model from different angles.

These experiments are also a proof of reliability. Since the missions were performed at depth of 28 m, it was not possible to use a buoy with a high bandwidth Wi-Fi connection. Only acoustic communication was available during the experiments.

### 5.4. Quantitative Evaluation

**Figure 24:**
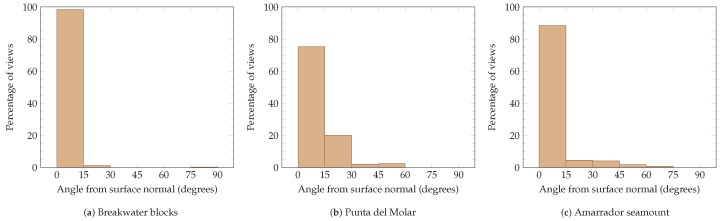
Histograms of the angles between the surface normal and the observation angle for all scenarios (**a**–**c**). Most viewed cells have been observed from a direction close to the surface normal.

As stated in Section 3, the viewpoints are placed so that images are obtained along the direction of the surface normal (small incidence angle). After all datasets have been acquired, the incidence angle has been evaluated in an offline procedure. Figure 24 represents the distribution of the best incidence angle for each viewed cell in the final map. In the breakwater blocks scenario, 98% of the viewed cells had been imaged with an incidence angle between 0 and 15 degrees. This measure decreases for environments with higher geometrical complexity. In the Punta del Molar, 75% of the viewed cells were imaged with an incidence angle between 0 and 15 degrees, and for the Amarrador seamount this measure increases to 88%. It is also important to remark that in all scenarios, more than 95% of the viewed cells have been observed within ±5% degrees from the central part of the camera’s FOV.

**Figure 25:**
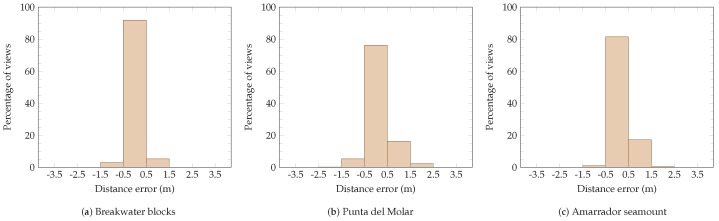
Histograms of the distance errors (distance between the target distance δ and the best observation distance) for all scenarios (**a**–**c**). Most viewed cells have been observed from a distance close to the desired distance.

The distance from which each viewed cell has been observed has also been analyzed. Figure 25 shows histograms of the distance error for each scenario. In the breakwater blocks, 92% of the viewed cells were imaged from a distance within 0.5 m from the target distance. For the Punta del Molar and Amarrador scenarios, this value is 76% and 81%, respectively.

## 6. Conclusions and Further Work

In this work we have presented a 2D frontier-based viewpoint generation algorithm for exploration using AUVs. While most of the existing underwater literature is focused on CPP algorithms, where previous information such as a rough map is used to plan coverage trajectories, our proposal does not require prior information and it is able to explore unknown 2D environments with elements of high relief.

The main contributions of this work are: (1) A novel 2D exploration algorithm which accounts for occupancy and optical data coverage simultaneously. (2) The combination of FB and view planning ideas in a single algorithm while keeping the computational requirements low. (3) Experimental evaluation through different sea trials, including a the breakwater concrete blocks, the Punta del Molar and the Amarrador scenarios, also showing a possible application such as 3D seabed reconstruction.

Further work will focus on finding an exploration strategy for the case where no initial viewpoints can be generated. We also plan to extend the algorithm to 3D environments, where we will use a multibeam sensor mounted in a tilting device on the Girona 500 AUV (see Ribas et al. [33]). Additionally, we would also like to expand the algorithm to be able to take into account viewpoints for multiple cameras. Our robotic exploration system would also benefit from a SLAM back-end to correct the drift present in the dead reckoning navigation of our vehicle. In this regard, Guillem et al. [34] has already used datasets, obtained with the previous version of the presented approach, to test a SLAM back-end. Having live feedback from the cameras would also open new possibilities for active localization/navigation and SLAM. Finally, modeling the uncertainty in the environment with probabilistic methods could be useful to improve the consistency of the map and the generation of next best viewpoints. 

## Figures and Tables

**Table 1:** Summary of the state of the art. The algorithms are classified by the amount of *prior knowledge* used, *domain*, *dimensionality* and *approach*.

Category	Domain	Space	Reference	Approach	Remarks
With prior map	Underwater	2.5D	Galceran et al. [10]	CPP and horizontal profiles	The terrain is classified in regions of low and high relief. The offline mission is adapted online using stochastic trajectory optimization
		3D	Palomeras et al. [11]	VP	A minimum set of views and TSP is used togenerate exploration trajectory, followed using SLAM. Simulation only
	Terrestrial	2D/3D	Blaer and Allen [12]	VP	Two stages. First, minimum set of views and TSP in 2D. Then, NBV in 3D
	Aerial	3D	Bircher et al. [13]	VP	Iterative viewpoint resampling with TSP in 3D
Without prior map	Underwater	2D	Williams et al. [14]	VP	Automatic target reinspection after an initial constant altitude mission
			Vidal et al. [8], Vidal et al. [9]	VP	Our previous work. Views are planned according to several frontiers
		3D	Kim and Eustice [15], Hover et al. [1]	VP	Perception driven navigation for the ship’s hull without prior map. Minimum set of views and TSP using a prior map for the propellers
			McEwen et al. [6]	RA	The 3D map is obtained by performing wall following at different depths
	Object reconstruction	3D	Connolly [5]	VP	Original proposal of the next-best-view (NBV) approach
			Vasquez-Gomez et al. [16], Vasquez-Gomez et al. [17]	FB and VP	It uses the frontiers to plan the NBV. Uncertainty is taken into account. Position and maximum size of the object must be known
			Isler et al. [18]	FB and VP	Information gain is used to plan the NBV. Position and maximum size of the object must be known
	Terrestrial	2D	Yamauchi [4]	FB	Original proposal of the FB approach. It clusters the frontier cells
			González-Baños and Mao [19]	VP	It builds a polygonal model of the environment and plans the NBV using a randomized algorithm that maximizes the information gain
			Burgard et al. [20]	FB	Multirobot exploration. Each robot is equipped with a 360 degree range sensor
			Fox et al. [21]	FB and VP	Multirobot exploration. Shared maps. The robots actively seek to verify their relative locations
			Stachniss et al. [22]	FB	Multirobot exploration. A classifier assigns labels to different locations in the map, and these labels are used in the utility function that guides the exploration
			Renzaglia and Martinelli [7]	RA	Potential fields are used to guide the exploration of a team of robots
	Aerial	3D	Schmid et al. [23]	VP	Viewpoints are planned using a coarse digital surface (DSM) in 2.5D. The data acquired from the viewpoints is used to create a 3D reconstruction
			Yoder and Scherer [24]	FB and VP	The exploration utility function is based on the visibility of 2D frontiers on the 2D surface of a 3D object
			Bircher et al. [25], Papachristos et al. [26]	Random tree and VP	A random tree is generated where the nodes are evaluated according to the amount of unmapped space that it explores

## Abbreviations

The following abbreviations are used in this manuscript:

2D	2-dimensional
2.5D	2.5-dimensional
3D	3-dimensional
AGP	art gallery problem
AHRS	attitude and heading reference system
AUV	autonomous underwater vehicle
AUVs	autonomous underwater vehicles
CIRS	underwater robotics research center
CPP	coverage path planning
DOF	degree of freedom
DOFs	degrees of freedom
DVL	Doppler velocity log
FB	frontier-based
FOV	field of view
FOVs	fields of view
GPS	global positioning system
ICS	inevitable collision state
ICSs	inevitable collision states
LOS	line of sight
MAV	micro aerial vehicle
MAVs	micro aerial vehicles
NBV	next-best-view
OMPL	open motion planning library
PDN	perception-driven navigation
PID	proportional-integral-derivative
RA	reactive algorithm
RAs	reactive algorithms
ROS	robot operating system
ROV	remotely operated vehicle
ROVs	remotely operated vehicles
RIG	rapidly-exploring information gathering
RRT	rapidly-exploring random tree
RRT*	asymptotic optimal rapidly-exploring random tree
SAS	synthetic aperture sonar
SLAM	simultaneous localization and mapping
TSP	traveling salesman problem
UAV	unmanned aerial vehicle
UAVs	unmanned aerial vehicles
UdG	university of Girona
UGV	unmanned ground vehicle
UGVs	unmanned ground vehicles
USBL	ultra-short baseline
VICOROB	computer vision and robotics group
VP	view planning
